# The effect of vitamin E supplementation on selected inflammatory biomarkers in adults: a systematic review and meta-analysis of randomized clinical trials

**DOI:** 10.1038/s41598-020-73741-6

**Published:** 2020-10-14

**Authors:** Omid Asbaghi, Mehdi Sadeghian, Behzad Nazarian, Mehrnoosh Sarreshtedari, Hassan Mozaffari-Khosravi, Vahid Maleki, Mohammad Alizadeh, Azad Shokri, Omid Sadeghi

**Affiliations:** 1 Nutritional Health Research Center, Lorestan University of Medical Sciences, Khorramabad, Iran; 2 Student Research Committee, Ahvaz Jundishapur University of Medical Sciences, Ahvaz, Iran; 3 Department of Nutrition, School of Allied Medical Sciences, Ahvaz Jundishapur University of Medical Science, Ahvaz, Iran; 4 Student Research Committee, Tabriz University of Medical Sciences, Tabriz, Iran; 5 Department of Nutrition, School of Public Health, Shahid Sadoughi University of Medical Sciences, Yazd, Iran; 6 Department of Clinical Nutrition, Faculty of Nutrition and Food Sciences, Tabriz University of Medical Sciences, Tabriz, Iran; 7 Nutrition Research Center, Faculty of Nutrition and Food Sciences, Tabriz University of Medical Sciences, Tabriz, Iran; 8 Social Determinants of Health Research Center, Kurdistan University of Medical Sciences, Sanandaj, Iran; 9 Gerash University of Medical Sciences, Gerash, Iran; 10 Students’ Scientific Research Center, Tehran University of Medical Sciences, Tehran, Iran; 11 Department of Community Nutrition, School of Nutritional Sciences and Dietetics, Tehran University of Medical Sciences, P.O. Box 14155-6117, Tehran, Iran

**Keywords:** Physiology, Biomarkers, Diseases, Medical research

## Abstract

The previous meta-analysis of clinical trials revealed a beneficial effect of vitamin E supplementation on serum C-reactive protein (CRP) concentrations; however, it is unknown whether this vitamin has the same influence on other inflammatory biomarkers. Also, several clinical trials have been published since the release of earlier meta-analysis. Therefore, we aimed to conduct a comprehensive meta-analysis to summarize current evidence on the effects of vitamin E supplementation on inflammatory biomarkers in adults. We searched the online databases using relevant keywords up to November 2019. Randomized clinical trials (RCTs) investigating the effect of vitamin E, compared with the placebo, on serum concentrations of inflammatory cytokines were included. Overall, we included 33 trials with a total sample size of 2102 individuals, aged from 20 to 70 years. Based on 36 effect sizes from 26 RCTs on serum concentrations of CRP, we found a significant reduction following supplementation with vitamin E (− 0.52, 95% CI − 0.80, − 0.23 mg/L, *P* < 0.001). Although the overall effect of vitamin E supplementation on serum concentrations of interleukin-6 (IL-6) was not significant, a significant reduction in this cytokine was seen in studies that used α-tocopherol and those trials that included patients with disorders related to insulin resistance. Moreover, we found a significant reducing effect of vitamin E supplementation on tumor necrosis factor-α (TNF-α) concentrations at high dosages of vitamin E; such that based on dose–response analysis, serum TNF-α concentrations were reduced significantly at the dosages of ≥ 700 mg/day vitamin E (P_non-linearity_ = 0.001). Considering different chemical forms of vitamin E, α-tocopherol, unlike other forms, had a reducing effect on serum levels of CRP and IL-6. In conclusion, our findings revealed a beneficial effect of vitamin E supplementation, particularly in the form of α-tocopherol, on subclinical inflammation in adults. Future high-quality RCTs should be conducted to translate this anti-inflammatory effect of vitamin E to the clinical setting.

## Introduction

Vitamin E is the most abundant lipid-soluble antioxidant present in body tissues^1^. Intake of the antioxidant has a beneficial effect on the prevention and management of chronic diseases including stroke, hypertension, diabetes mellitus, and fatty liver disease^2,3^. Subclinical inflammation is a low-grade chronic inflammation with only minor elevation in C-reactive protein (CRP) levels occurring in the absence of classic clinical signs of inflammation. The major function of such inflammation is to restore tissue homeostasis^4–6^. Whether the beneficial effect of vitamin E on these chronic conditions is mediated through the inflammatory processes is not clear. It has been shown that vitamin E has an inhibitory effect on pro-inflammatory cytokine expression through inhibition of activation of the key nuclear transcription factor NF-κB^7,8^. Also, there is evidence from observational studies indicating an overall favorable link between vitamin E intake and serum levels of pro-inflammatory cytokines^9,10^. Despite the aforesaid points, findings from clinical trials investigating the effect of vitamin E supplementation on subclinical inflammation are conflicting^11–43^. Some clinical trials have shown a significant reducing effect of vitamin E supplementation on serum concentrations of C-reactive protein (CRP) and interleukin-6 (IL-6)^26,37,40^, while others did not find any significant effect^17,19,27^. Surprisingly, in a randomized clinical trial (RCT), vitamin E supplementation resulted in a significant increase in serum concentrations of CRP and IL-6^33^. An earlier meta-analysis of vitamin E supplementation on serum CRP levels suggested a beneficial effect of vitamin E in the form of either α-tocopherol or γ-tocopherol^44^. Also, a meta-analysis in 2014 revealed a beneficial effect of vitamin E coated dialyzer on subclinical inflammation; however, authors in that meta-analysis included only clinical trials that performed on hemodialysis patients^45^.

Overall, there is a need for a comprehensive meta-analysis summarizing all available findings in this area. Therefore, the current meta-analysis was conducted to summarize current evidence on the effects of vitamin E supplementation on inflammatory biomarkers in adults.

## Methods

This study was performed based on the PRISMA (Preferred Reporting Items for Systematic Reviews and Meta-Analyses) protocol for reporting systematic reviews and meta-analyses.

### Search strategy

We performed a comprehensive literature search in the online databases of PubMed, Scopus, Web of Science, and Google Scholar up to November 2019. In the search, we purposed to identify clinical trials investigating the effects of vitamin E supplementation on inflammatory cytokines in adults. The following keywords were used in the search strategy: ("vitamin E" OR tocopherol OR tocotrienol OR "VIT E") AND ("Inflammation" OR "inflammatory " OR "Interleukin-10" OR IL-10 OR "Interleukin-8" OR IL-8 OR "Interleukin-6" OR IL-6 OR "Tumor necrosis factor" OR TNF OR "C-reactive protein" OR " high-sensitivity c-reactive protein" OR CRP OR hs-CRP OR "Transforming growth factor beta" OR "cytokines" OR "cytokine" OR "Acute phase reactant" OR "Matrix metalloproteinase" OR "e-selectin" OR "p-selectin" OR "Intercellular adhesion molecule-1" OR "Monocyte chemotactic protein 1" OR MCP-1 OR "Inflammation Mediator" OR "Neurogenic Inflammation" OR "Myokine" OR "Adipokine" OR "Interleukin-1B" OR IL-1B OR "interleukins" OR "interleukin" OR "Systemic inflammation" OR "Biological marker" OR "Fibrinogen”). No language or time restriction was applied. Reference lists of the relevant studies were manually screened to avoid missing any eligible publication. Unpublished studies were not considered. The literature search was conducted by two independent investigators.

### Inclusion criteria

We included eligible studies that met the following criteria: (1) randomized controlled clinical trials, (2) studies that conducted on adult subjects (≥ 18 years), (3) studies that administered vitamin E in different chemical forms including alpha-, beta-, gamma-, and delta-tocopherol and alpha-, beta-, gamma-, and delta-tocotrienol, (4) RCTs with at least one week’s duration of intervention, and (5) controlled trials that reported mean changes and their standard deviations (SDs) of inflammatory cytokines throughout the trial for both intervention and control groups or presented required information for calculation of those effect sizes. If > 1 article were published for one dataset, the more complete one was included. Clinical trials with an additional intervention group were considered as 2 separate studies.

### Exclusion criteria

In the current meta-analysis, we excluded experimental studies, those with a cohort, cross-sectional, and case–control design, review articles, and ecological studies. We also excluded trials without a placebo or control group and those which were performed on children or adolescents.

### Data extraction

Two independent investigators performed data extraction from each eligible RCT. The following information was extracted: name of the first author, publication year, individuals’ characteristics (mean age and sex), study design, sample size (control and intervention groups), type of vitamin E prescribed, the dosage of vitamin E, duration of intervention, mean changes and their SDs of inflammatory biomarkers throughout the trial for the intervention and control groups, and the confounding variables adjusted in the analyses. If data on inflammatory biomarkers were reported in different units, we converted them to the most frequently used unit.

### Risk of bias assessment

We applied the Cochrane quality assessment tool for assessing the risk of bias for each study included in the current meta-analysis^46,47^. This tool contained seven domains including random sequence generation, allocation concealment, reporting bias, performance bias, detection bias, attrition bias, and other sources of bias. Each domain was given a “high risk” score if the study comprised methodological defects that may have affected its findings, a “low risk” score if there was no defect for that domain, and an “unclear risk” score if the information was not sufficient to determine the impact. The overall risk of bias for an RCT was considered: (1) Low; if all domains had “low risk”, (2) Moderate; if one or more domains had “unclear risk”, and (3) High; if one or more domains had “high risk”^48^. The risk of bias assessment was done independently by two reviewers.

### Statistical analysis

Mean changes and their SDs of inflammatory cytokines in the vitamin E and control groups were used to obtain the overall effect sizes. When mean changes were not reported, we calculated them by considering changes in cytokine concentrations during the intervention. We also converted standard errors (SEs), 95% confidence intervals (CIs), and interquartile ranges (IQRs) to SDs using the method of Hozo et al.^49^*.* To obtain the overall effect sizes, we applied a random-effects model that takes between-study variations into account. Heterogeneity was determined by the I^2^ statistic and Cochrane’s Q test. I^2^ value > 50% or P < 0.05 for the Q-test was considered as significant between-study heterogeneity^50–52^. To find probable sources of heterogeneity, subgroup analyses were performed according to the predefined variables including duration of the intervention (≥ 8 vs. < 8 weeks), type (alpha-tocopherol vs. gamma-tocopherol vs. mixed type) and dosage of vitamin E (≥ 500 vs. < 500 mg/day), participants’ health condition (apparently healthy vs. unhealthy individuals), baseline serum levels of inflammatory cytokines (elevated vs. normal levels), and adjustment for baseline levels of the outcome variable (adjusted vs. non-adjusted). To determine the non-linear effects of vitamin E dosage (mg/d) on cytokine concentrations, fractional polynomial modeling was applied. Sensitivity analysis was used to detect the dependency of the overall effect size on a particular study. The possibility of publication bias was examined by the formal test of Begg. The meta-analysis was carried out by the use of the Stata, version 11.2 (StataCorp). *P-*value < 0.05 was considered as significant level.

## Results

**Figure 1:**
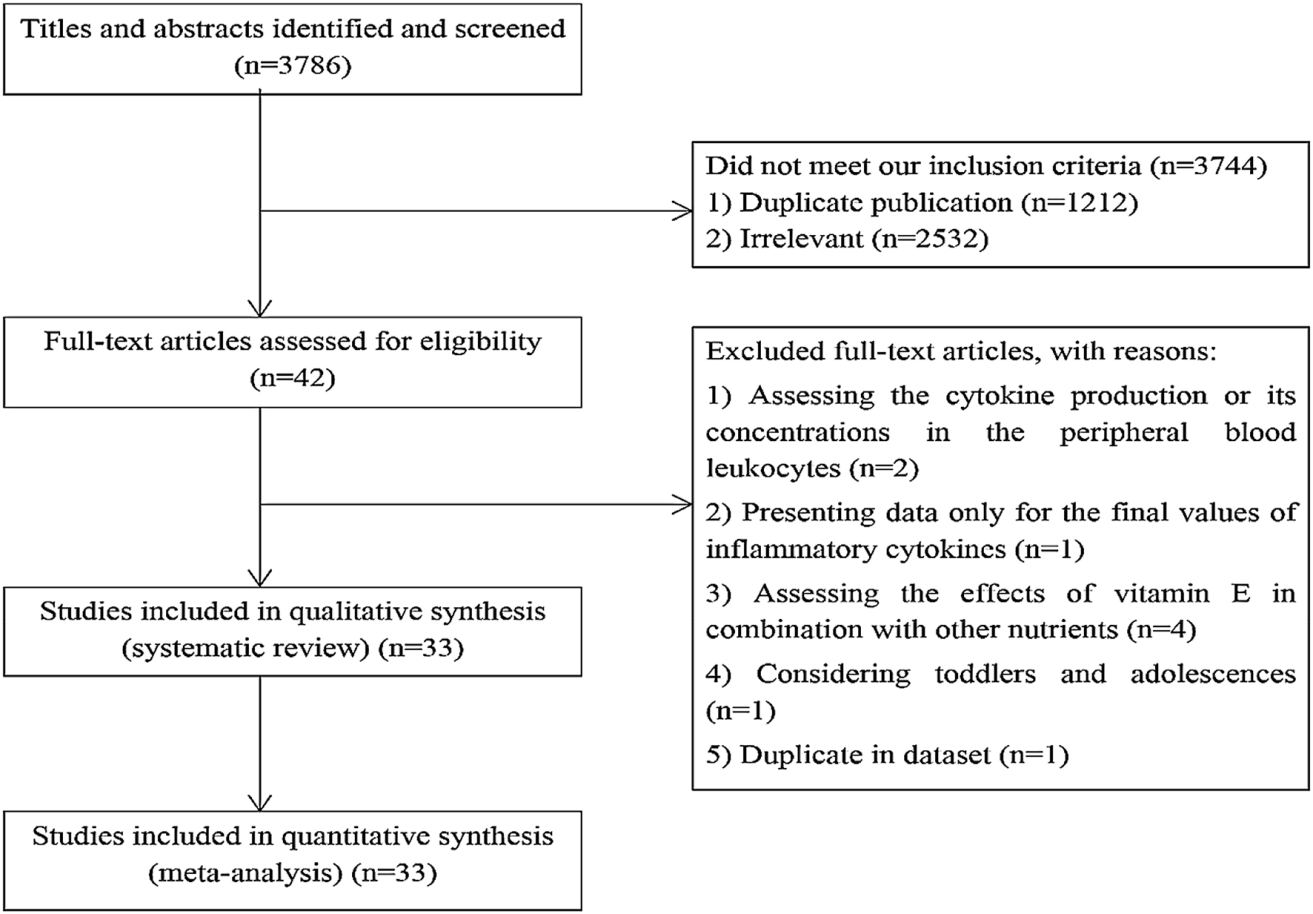
Flow diagram of the study selection.

Out of 3786 publications that were identified in our initial search, 1212 duplicate articles were excluded. After screening the remaining 2574 records, 2532 unrelated articles were also removed based on title and abstract assessment. Then, 42 publications remained for further evaluation of the full text. Out of those 42 studies, two RCTs were excluded due to assessing the cytokine production or its concentrations in the peripheral blood leukocytes^53,54^. The study of Silva et al. was also excluded because they did not report baseline values of cytokine concentrations making us unable to calculate mean changes of cytokines throughout the trial^55^. We also excluded four RCTs in which the effects of vitamin E in combination with other nutrients such as vitamin C, α-lipoic acid, and omega-3 fatty acids were investigated^56–59^. The study of Tang et al. performed on iron-deficient infants and toddlers was excluded as well^60^. Moreover, two eligible articles were published on the same dataset^35,61^, of which the more complete one was included^35^ and the other one was excluded^61^. After these exclusions, 33 eligible RCTs remained for inclusion in the current systematic review and meta-analysis^11–43^; out of them, 26 studies assessed serum concentrations of CRP^11,13–20,22,25–31,33–35,37,38,40–43^, 14 studies assessed serum concentrations of IL-6^17,20–23,26–31,33,37,38^, and 12 studies evaluated serum concentrations of tumor necrosis factor-α (TNF-α) following vitamin E supplementation^12,17,20,21,24,29–32,36,38,39^. Data on the other inflammatory cytokines including IL-1 (n = 1), IL-2 (n = 2), IL-4 (n = 1), and IL1β (n = 1) were not sufficient to perform a meta-analysis. Flow diagram of study selection is outlined in Fig. 1.

### Characteristics of the included studies

The characteristics of 33 RCTs included in the current systematic review and meta-analysis are illustrated in Table 1. These RCTs were published between 2000 and 2018 and were from the USA^14,20,27,29,42,43^, Europe^13,15,19,21,22^, Asia^11,18,23–26,28,30–37,39–41^, Canada^12^, and Australia^16,17,38^. Four studies were exclusively performed on male subjects^23,24,28,35^ and others on both genders. The sample size of included RCTs varied from 16 to 110 participants, resulting in a total sample size of 2102 individuals. The mean age of participants was between 20 and 70 years. The dosage of vitamin E supplements varied from 15 to 1080 mg/day and duration of intervention ranged from 1 to 104 weeks across selected RCTs. Most studies employed a parallel design, while only one study was cross-over^22^. Concerning the type of vitamin E, a total of 27 studies administered α-tocopherol^11–15,17–26,28,30–37,39,42,43^, 3 studies administered γ-tocopherol^16,20,29^, and 3 other studies performed the intervention with a combination of different types of tocopherols. In six trials, participants in the vitamin E and control groups received concentrated red grape juice^19^ or supplements of α-lipoic acid^26,30^ or n-3 fatty acids^14,18,32^ in addition to the main intervention. RCTs were performed on healthy individuals^14,16,23,24,29^, patients with type 2 diabetes^11,17,25,31,33,36,38^, metabolic syndrome^20,30^, non-alcoholic fatty liver disease^21,39,40^, cardiovascular diseases (CVDs)^12,13,22,35,41,42^, rheumatoid arthritis^18,32^, erectile dysfunction^28^, and renal calculi^34^. Furthermore, five trials included hemodialysis patients^15,19,26,37,43^. Of 33 RCTs, 11 studies controlled the baseline values of inflammatory cytokines in their analyses^11,16–19,25,36,37,39,40,43^. Only one study^30^ could be considered as a high-quality study with a totally low risk of bias for all domains of the Cochrane Risk of Bias Assessment Tool. Two RCTs^29, 38^ were of moderate-quality in which one or more domains had an unclear risk of bias. Others had low-quality since they had a high risk of bias for one or more domains (Supplemental Table 1).

**Table 1:** Summary of clinical trials on the effects of vitamin E supplementation on inflammatory biomarkers in adults aged ≥ 20 years.

Author, year	Design	Participants, n	Health condition	Age, year^a^	Intervention		Duration (week)	Outcomes (changes)^b^		Adjust/matching^c^
					Treatment group	Control group		Treatment group	Control group	
Upritchard et al. 2000	RA/parallel	M/F: 25 Int: 12, Con: 13	DM	Int: 56 ± 14, Con: 60 ± 6	800 IU/day α-tocopherol		4	CRP: − 2.70 ± 3.70	CRP: 0.20 ± 1.75	4
Keith et al. 2001	RA/DB/parallel	M/F: 56 Int: 30, Con: 26	CVD	Int: 70, Con: 64	1000 IU/day α-tocopherol		12	TNF-α: 0.40 ± 2.22	TNF-α: 0.99 ± 2.65	
Murphy et al. 2004	RA/DB/parallel	M/F: 110 Int: 55, Con: 55	CVD	Int: 65 ± 9, Con: 66 ± 13	400 IU/day α-tocopherol acetate		26	CRP: − 10.47 ± 10.48	CRP: − 14.19 ± 33.09	
Lopez et al. 2004	RA/DB/parallel	M/F: 40 Int: 20, Con: 20	Healthy	Int: 33 ± 7, Con: 33 ± 10	800 IU/day all-rec α-tocopherol		12	CRP: 0.10 ± 4.75	CRP: 0. ± 1.61	
		M/F: 40 Int: 20, Con: 20		Int: 29 ± 6, Con: 30 ± 9	800 IU/day all-rec α-tocopherol + 1.5 g n-3 PUFA			CRP: 0.40 ± 4.79	CRP: 1.10 ± 6.28	
Hodkova et al. 2006	RA/parallel	M/F: 29 Int: 15, Con: 14	HD	Int: 63 ± 6, Con: 60 ± 8	400 mg/day α-tocopherol		5	CRP: − 0.72 ± 2.56	CRP: 1.13 ± 3.28	1
Singh et al. 2007	RA/DB/parallel	M/F: 26 Int: 14, Con: 12	Healthy	Int: 20–40, Con: 20–40	100 mg/day γ-tocopherol		5	CRP: 0.50 ± 2.76	CRP: − 0.30 ± 2.10	2,4
		M/F: 25 Int: 13, Con: 12			200 mg/day γ-tocopherol			CRP: − 0.40 ± 1.45	CRP: − 0.30 ± 2.10	
Wu et al. 2007	RA/DB/parallel	M/F: 36 Int: 18, Con: 18	DM	Int: 64 ± 30, Con: 62 ± 30	500 mg/day α-tocopherol		6	CRP: 0.02 ± 1.34 TNF-α: 0.03 ± 0.15 IL-6: − 0.33 ± 0.65	CRP: − 0.20 ± 1.46 TNF-α: 0.03 ± 0.19 IL-6: 0.15 ± 0.71	4
		M/F: 37 Int: 19, Con: 18		Int: 58 ± 17, Con: 62 ± 30	500 mg/day mixed tocopherols			CRP: − 0.21 ± 0.79 TNF-α: 0.02 ± 0.13 IL-6: 0.05 ± 0.45	CRP: − 0.20 ± 1.46 TNF-α: 0.03 ± 0.19 IL-6: 0.15 ± 0.71	
Aryaeian et al. 2008	RA/DB/parallel	M/F: 43 Int: 21, Con: 22	rheumatoid arthritis	Int: 49 ± 12, Con: 48 ± 11	400 mg/day α-tocopherol		12	CRP: − 4.99 ± 11.00	CRP: − 0.96 ± 4.77	4,9
		M/F: 44 Int: 22, Con: 22		Int: 44 ± 13, Con: 46 ± 3	400 mg/day α-tocopherol + CLA	CLA		CRP: − 2.06 ± 4.04	CRP: − 1.72 ± 6.60	
Castilla et al. 2008	RA/parallel	M/F: 16 Int: 8, Con: 8	HD	33–79	800 IU/day α-tocopherol	Nothing	2	CRP: 3.50 ± 8.44	CRP: 0.69 ± 6.36	4
		M/F: 16 Int: 8, Con: 8			800 IU/day α-tocopherol plus red grape juice	Red grape juice		CRP: − 1.10 ± 6.46	CRP: 2.40 ± 10.78	
Devaraj et al. 2008	RA/DB/parallel	M/F: 40 Int: 20, Con: 20	MetS	Int: 51 ± 11, Con: 56 ± 11	800 mg/day α-tocopherol		6	CRP: − 0.86 ± 3.15 TNF-α: − 0.04 ± 0.21 IL-6: 0.20 ± 1.46	CRP: − 0.38 ± 3.29 TNF-α: 0.08 ± 0.29 IL-6: 0.50 ± 1.64	1,3
		M/F: 40 Int: 20, Con: 20		Int: 50 ± 9, Con: 56 ± 11	800 mg/day γ-tocopherol			CRP: − 1.49 ± 2.80 TNF-α: − 0.02 ± 0.27 IL-6: − 0.30 ± 1.02	CRP: − 0.38 ± 3.29 TNF-α: 0.08 ± 0.29 IL-6: 0.50 ± 1.64	
		M/F: 40 Int: 20, Con: 20		Int: 57 ± 14, Con: 56 ± 11	800 mg/day mixed tocopherols			CRP: − 2.20 ± 3.05 TNF-α: − 0.14 ± 0.30 IL-6: 0.30 ± 1.26	CRP: − 0.38 ± 3.29 TNF-α: 0.08 ± 0.29 IL-6: 0.50 ± 1.64	
Balmer et al. 2009	RA/DB/parallel	M/F: 28 Int: 14, Con: 14	NASH	Int: 47 ± 14, Con: 47 ± 12	800 IU/day α-tocopherol		104	TNFα: − 0.75 ± 0.66 IL-6: − 0.47 ± 2.44	TNFα: − 1.65 ± 0.85 IL-6: 0.69 ± 1.88	1
Dalgard et al. 2009	RA/DB/crossover	M/F: 48 Int: 24, Con: 24	CVD	Int: 63 ± 7, Con: 57 ± 6	15 mg/day α-tocopherol + fruits juice	Fruits juice	4	CRP: − 0.20 ± 0.74 IL-6: 0.10 ± 0.81	CRP: − 0.10 ± 2.00 IL-6: − 0.10 ± 0.96	
Ghiasvand et al. 2009	RA/DB/parallel	M: 17 Int: 9, Con: 8	Healthy	Int: 24 ± 2, Con: 21 ± 2	400 IU/day α-tocopherol		6	IL-6: − 0.10 ± 1.27	IL-6: 0.16 ± 1.43	1,2
		M: 17 Int: 9, Con: 8		Int: 27 ± 5, Con: 24 ± 3	400 IU/day α-tocopherol + EPA	EPA		IL-6: − 2.83 ± 1.49	IL-6: − 3.81 ± 0.94	
Ghiasvand et al. 2010	RA/DB/parallel	M: 17 Int: 9, Con: 8	Healthy	Int: 23 ± 2, Con: 21 ± 2	400 IU/day α-tocopherol		6	TNF-α: 1.44 ± 3.60	TNF-α: 0.37 ± 3.62	1,2
		M: 17 Int: 9, Con: 8		Int: 27 ± 5, Con: 24 ± 3	400 IU/day α-tocopherol + EPA	EPA		TNF-α: 3.50 ± 2.40	TNF-α: 0.88 ± 4.45	
Rafraf et al. 2012	RA/DB/parallel	M/F: 83 Int: 42, Con: 41	DM	Int: 35 ± 7, Con: 35 ± 8	400 mg/day α-tocopheryl acetate		8	CRP: − 0.03 ± 0.59	CRP: 0.07 ± 0.79	1,2,3,4,6,8
Ahmadi et al. 2013	RA/parallel	M/F: 41 Int: 17, Con: 24	HD	Int: 45 ± 13, Con: 49 ± 12	400 IU/day α-tocopherol		8	CRP: − 2.00 ± 5.17 IL-6: − 10.00 ± 20.26	CRP: 0.19 ± 3.92 IL-6: 10.9 ± 25.50	-
		M/F: 44 Int: 24, Con: 20		Int: 53 ± 10, Con: 49 ± 11	400 IU/day α-tocopherol + lipoic acid	lipoic acid		CRP: − 1.60 ± 4.08 IL-6: − 11.00 ± 20.13	CRP: − 2.50 ± 4.71 IL-6: − 7.50 ± 17.15	
Daud et al. 2013	RA/DB/parallel	M/F: 81 Int: 41, Con: 40	HD	Int: 59 ± 12, Con: 58 ± 13	220 mg/day tocotrienol-rich fraction, all types		16	CRP: 1.30 ± 16.90 IL-6: 1.00 ± 2.12	CRP: 1.30 ± 23.86 IL-6: 0.60 ± 4.40	
El-sisi et al. 2013	RA/DB/parallel	M: 40 Int: 20, Con: 20	Erectile dysfunction	40–60	400 IU/day α-tocopherol		6	CRP: 0.66 ± 2.55 IL-6: − 3.17 ± 2.32	CRP: 0.17 ± 1.74 IL-6: 0.79 ± 2.67	
Mah et al. 2013	RA/DB/parallel	M/F: 30 Int: 16, Con: 14	Healthy smokers	Int: 21 ± 4, Con: 22 ± 4	500 mg/day γ-tocopherol		1	CRP: − 1.73 ± 5.28 TNF-α: − 0.29 ± 0.40 IL-6: − 0.02 ± 0.48	CRP: − 0.96 ± 2.31 TNF-α: − 0.05 ± 0.2 IL-6: − 0.38 ± 0.80	
Manning et al. 2013	RA/DB/parallel	M/F: 76 Int: 36, Con: 40	MetS	Int: 57 ± 10, Con: 57 ± 9	100 IU/day α-tocopherol		52	CRP: 0.50 ± 2.42 TNFα: − 0.09 ± 0.44 IL-6: − 0.30 ± 0.44	CRP: 0.00 ± 2.01 TNFα: 0.00 ± 0.32 IL-6: 0.10 ± 0.79	2
		M/F: 75 Int: 41, Con: 34		Int: 54 ± 13, Con: 55 ± 10	100 IU/day α-tocopherol + lipoic acid	Lipoic acid		CRP: − 0.20 ± 2.75 TNFα: 0.30 ± 0.40 IL-6: 0.40 ± 1.07	CRP: 0.30 ± 1.68 TNFα: 0.00 ± 0.44 IL-6: 0.99 ± 0.93	
Shadman et al. 2013	RA/DB/parallel	M/F: 36 Int: 17, Con: 19	Overweight DM	Int: 48 ± 4, Con: 45 ± 6	100 IU/day α-tocopherol		8	CRP: − 0.98 ± 2.71 TNFα: − 2.80 ± 1.83 IL-6: − 0.60 ± 1.83	CRP: − 0.55 ± 1.58 TNFα: − 3:00 ± 1.50 IL-6: − 0.89 ± 0.98	1,2,3
Aryaeian et al. 2014	RA/DB/parallel	M/F: 43 Int: 21, Con: 22	rheumatoid arthritis	Int: 49 ± 12, Con: 48 ± 11	400 mg/day α-tocopherol		12	TNFα: − 1.17 ± 2.88	TNFα: − 1.13 ± 2.74	1,2,10
		M/F: 44 Int: 22, Con: 22		Int: 44 ± 13, Con: 46 ± 13	400 mg/day α-tocopherol + CLA			TNFα: − 2.41 ± 2.59	TNFα: − 2.36 ± 3.63	
Gopalan et al. 2014	RA/DB/parallel	M/F: 88 Int: 46, Con: 42	CVD	Int: 52 ± 9, Con: 52 ± 8	400 mg/day mixed tocotrienols		104	CRP: − 0.57 ± 4.27	CRP: 2.12 ± 10.76	
Hejazi et al. 2015	RA/SB/parallel	M/F: 27 Int: 14, Con: 13	DM	Int: 48 ± 62, Con: 47 ± 8	400 IU/day α-tocopherol		6	CRP: 1.30 ± 8.17 IL-6: 15.40 ± 8.96	CRP: − 0.70 ± 4.69 IL-6: 5.60 ± 3.83	
Modi et al. 2015	RA/parallel	M/F: 72 Int: 36, Con: 36	Renal calculi	Int: 39 ± 5, Con: 40 ± 4	800 mg/day α-tocopherol		1	CRP: 0.09 ± 1.40	CRP: 2.99 ± 4.28	
Ramezani et al. 2015	RA/DB/parallel	M/F: 42 Int: 20, Con: 22	CVD	Int: 56 ± 2, Con: 55 ± 1	400 IU/day α-tocopherol		8	CRP: − 1.57 ± 2.41	CRP: − 1.29 ± 1.99	
Khatami et al. 2016	RA/DB/parallel	M/F: 60 Int: 30, Con: 30	DM	Int: 61 ± 10, Con: 62 ± 14	1200 IU/day α-tocopherol		12	TNF-α: − 32.8 ± 24.90	TNF-α: 3.0 ± 24.09	1,2,3,4,6,7
Sohrabi et al. 2016	RA/parallel	M/F: 46 Int: 23, Con: 23	HD	Int: 56 ± 9, Con: 57 ± 10	1800 IU/week all-rec α-tocopherol + whey protein		8	CRP: − 0.98 ± 0.23 IL-6: − 1.18 ± 2.70	CRP: − 0.34 ± 0.87 IL-6: − 3.96 ± 14.25	4
		M/F: 46 Int: 23, Con: 23		Int: 58 ± 8, Con: 55 ± 6	1800 IU/week all-rec α-tocopherol	Nothing		CRP: 0.002 ± 0.90 IL-6: − 5.10 ± 17.90	CRP: 0.06 ± 0.34 IL-6: 2.77 ± 4.80	
Stonehouse et al. 2016	RA/DB/parallel	M/F: 57 Int: 28, Con: 29	DM	Int: 60 ± 7, Con: 61 ± 6	552 mg/day tocotrienol, all types		8	CRP: 0.38 ± 1.68 TNF-α: − 0.09 ± 1.32 IL-6: 0.37 ± 5.77	CRP: − 0.07 ± 1.52 TNF-α: − 0.4 ± 1.29 IL-6: − 2.13 ± 5.67	1,2,3,5,6
Ekhlasi et al. 2017	RA/DB/parallel	M/F: 30 Int: 15, Con: 15	NAFLD	25–64	400 IU/day α-tocopherol		8	TNF-α: − 11.66 ± 10.4	TNF-α: 2.57 ± 10.1	1,3,4
					400 IU/day α-tocopherol + probiotic strain	Probiotic strain		TNF-α: − 15.01 ± 10.0	TNF-α: − 9.1 ± 10.1	
Pervez et al. 2018	RA/DB/parallel	M/F: 64 Int: 31, Con: 33	NAFLD	Int: 45 ± 9, Con: 44 ± 10	600 mg/day δ-tocotrienol (90%) and γ-tocotrienol (10%)		12	CRP: − 0.74 ± 0.29	CRP: − 0.26 ± 0.30	4
Devaraj et al. 2007	RA/DB/parallel	M/F: 90 Int: 44, Con: 46	CVD	Int: 59 ± 7, Con: 62 ± 6	1200 IU/day α-tocopherol		104	CRP: − 1.69 ± 1.69	CRP: 0.61 ± 1.76	
Rachelle et al. 2011	RA/DB/parallel	M/F: 50 Int: 25, Con: 25	HD	Int: , Con:	400 IU/day α-tocopherol		8	CRP: − 5.80 ± 13.29	CRP: 6.40 ± 11.48	4

*CRP* C-reactive protein, *IL-6* interleukin 6, *TNF-α* tumour necrosis factor-α,* DM* diabetes mellitus, *CVD* cardiovascular disease, *NASH* nonalcoholic steatohepatitis, *NAFLD* non-alcoholic fatty liver disease, *HD* hemodialysis, *MetS* metabolic syndrome, *RA* randomized, *DB* double-blinded, *M* male, *F* female, *Int* intervention, *Con* control.

^a^Values are mean ± SD or range (for age).

^b^Changes in cytokine concentrations are presented by common units for CRP (mg/L), IL-6 (pg/mL) and TNF-α (pg/mL).

^c^Adjustment or matching: age (1), sex (2), BMI (3), baseline values of cytokines (4), DM (5), duration of DM (6), use of medication or supplements (7), dietary intake of vitamin E (8), changes in other variables (9), disease duration (10).

### Findings from the systematic review

Among 26 studies assessing the serum concentrations of CRP, 6 studies revealed a significant reducing effect of vitamin E supplementation on serum CRP concentrations^11,34,37,40,42,43^, whereas others found no significant effect. Four trials showed a significant reduction^26,28,30,37^ and one study indicated a significant increase in serum IL-6 concentrations^33^ following vitamin E supplementation, while others revealed no significant change. Of 12 RCTs that examined the effects of vitamin E supplementation on serum TNF-α concentrations, two studies reported a beneficial effect^36,39^, whereas two trials showed an increasing effect of vitamin E supplementation on serum concentrations of TNF-α^21,30^. The remaining studies on TNF-α reported no significant effect.

### Findings from the meta-analysis

Overall, 33 RCTs in the systematic review were included in the meta-analysis. These trials had a total sample size of 2102 individuals with the age of 20 years and over.

### The effect of vitamin E on serum CRP concentrations

**Figure 2:**
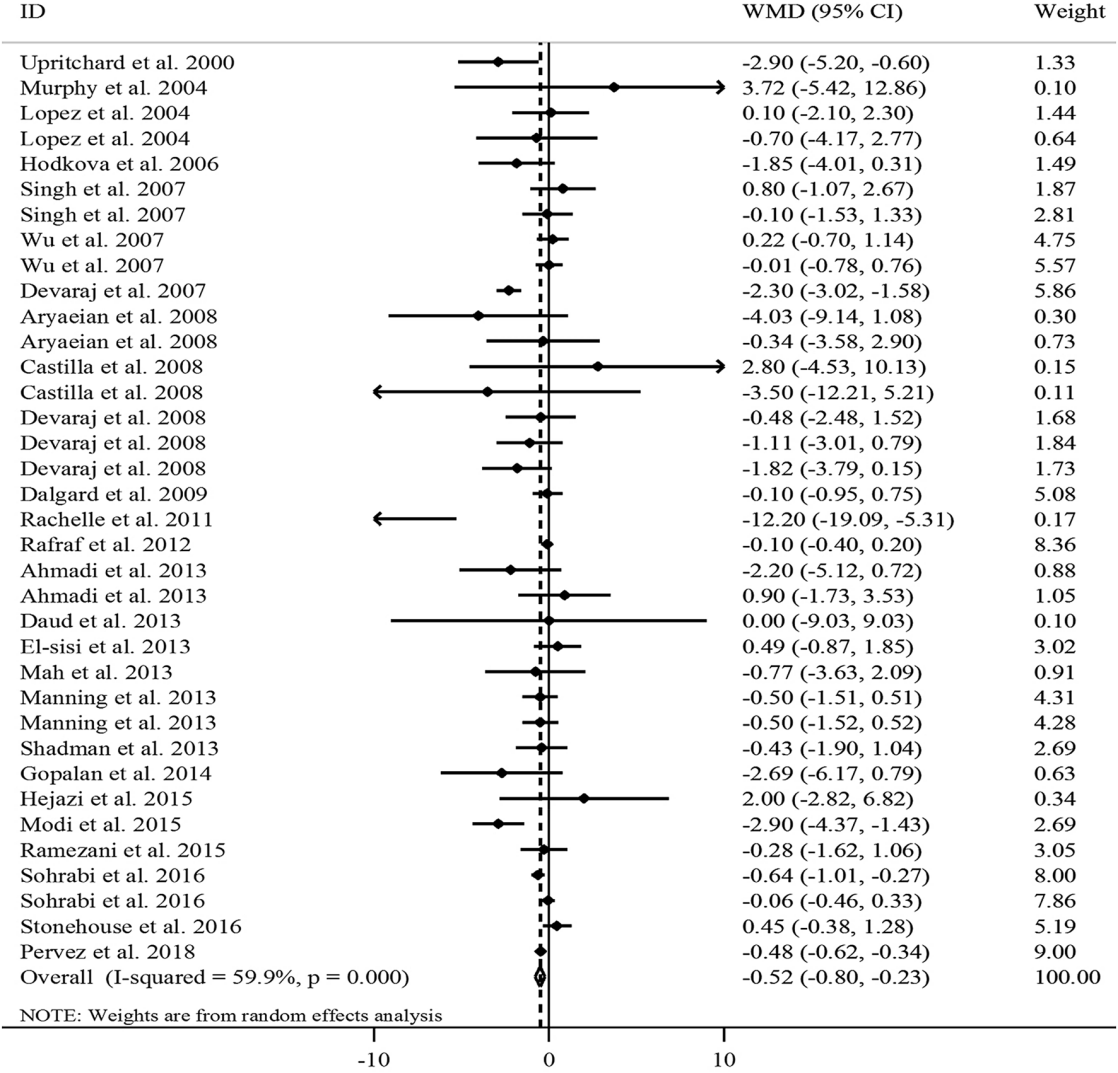
Forest plot for the effect of vitamin E supplementation on serum CRP concentrations, expressed as mean differences between intervention and control groups. Horizontal lines represent 95% CIs. Diamonds represent pooled estimates from the random-effects analysis. *CRP* C-reactive protein, *WMD* weighted mean difference, *CI* confidence interval.

In total, 26 RCTs with a total sample size of 1743 subjects were included in the analysis^11,13–20,22,25–31,33–35,37,38,40–43^. Combining 36 effect sizes from these studies indicated that vitamin E supplementation, compared with controls, resulted in a significant reduction in serum CRP concentrations [weighted mean difference (WMD) − 0.52, 95% CI − 0.80, − 0.23 mg/L, *P* < 0.001] (Fig. 2).

However, there was evidence of a moderate between-study heterogeneity (*I*^2^ = 59.9, P < 0.001).

To detect potential sources of heterogeneity, subgroup analyses were performed (Table 2). We found that the between-study heterogeneity was explained by the type and dosage of tocopherol, participants’ health condition, and baseline serum concentrations of CRP. From these analyses, we found a significant reducing effect of vitamin E supplementation on serum CRP concentrations in RCTs with intervention duration of ≥ 8 weeks, trials that administered α-tocopherol or a mixed type of tocopherols, and RCTs that prescribed ≥ 500 mg/day vitamin E. Besides, a significant reduction was observed in studies that were conducted on unhealthy participants including hemodialysis patients or subjects with CVDs and disorders related to insulin resistance, trials on individuals with normal or elevated levels of CRP, and studies that provided effect size whether adjusted or did not adjust for baseline serum concentrations of CRP. In the sensitivity analysis, exclusion of any single study did not affect the overall estimate for the effect of vitamin E supplementation on serum CRP concentrations (range of summary estimates: − 0.84, − 0.12). In addition, based on the Begg’s test, no evidence of substantial publication bias was seen (*P* = 0.20).

**Table 2:** Subgroup analyses for the effects of vitamin E supplementation on inflammatory biomarkers in adults aged ≥ 20 years.

	Effect size, *n*	WMD (95% CI)^a^	P-within^b^	*I*^2^ (%)^c^	P-heterogeneity^d^
**Vitamin E supplementation on serum CRP concentrations**					
Overall	36	− 0.43 (− 0.54, − 0.33)	< 0.001	59.9	< 0.001
Intervention duration (week)					
< 8	16	− 0.35 (− 0.72, 0.01)	0.059	47.6	0.018
≥ 8	20	− 0.44 (− 0.55, − 0.33)	< 0.001	67.5	< 0.001
Type of vitamin E					
α-Tocopherol	26	− 0.42 (− 0.59, − 0.25)	< 0.001	66.9	< 0.001
γ-Tocopherol	4	− 0.19 (− 1.11, 0.73)	0.685	0.0	0.542
Mixed-tocopherols	6	− 0.45 (− 0.59, − 0.31)	< 0.001	46.7	0.095
Dosage of vitamin E (mg/day)					
< 500	19	− 0.18 (− 0.42, 0.06)	0.135	29.0	0.115
≥ 500	17	− 0.50 (− 0.61, − 0.38)	< 0.001	71.8	< 0.001
Health condition					
Healthy	5	0.04 (− 0.88, 0.96)	0.933	0.0	0.885
Unhealthy	31	− 0.44 (− 0.55, − 0.33)	< 0.001	64.8	< 0.001
Insulin resistance-related disorders	13	− 0.39 (− 0.51, − 0.26)	< 0.001	39.4	0.071
CVDs	5	− 1.25 (− 1.75, − 0.75)	< 0.001	79.0	0.001
Hemodialysis	9	− 0.41 (− 0.67, − 0.15)	0.002	62.1	0.007
Rheumatoid arthritis	2	− 1.40 (− 4.13, 1.34)	0.317	30.0	0.232
Other disease	2	− 1.06 (− 2.06, − 0.07)	0.037	90.9	0.001
Baseline concentrations of CRP (mg/L)					
Normal (< 3 mg/L)	18	− 0.31 (− 0.51, − 0.10)	0.004	69.0	< 0.001
Elevated (≥ 3 mg/L)	18	− 0.48 (− 0.60, − 0.35)	< 0.001	44.4	0.022
Adjustment for baseline values					
Adjusted	14	− 0.39 (− 0.50, − 0.27)	< 0.001	60.5	0.002
Non-adjusted	22	− 0.76 (− 1.07, − 0.45)	< 0.001	57.6	< 0.001
**Vitamin E supplementation on serum IL-6 concentrations**					
Overall	21	− 0.14 (− 0.29, 0.01)	0.06	74.3	< 0.001
Intervention duration (week)					
< 8	11	− 0.12 (− 0.32, 0.07)	0.220	80.6	< 0.001
≥ 8	10	− 0.17 (− 0.40, 0.06)	0.145	65.7	0.002
Type of vitamin E					
α-Tocopherol	15	− 0.21 (− 0.39, − 0.03)	0.023	79.2	< 0.001
γ-Tocopherol	2	0.08 (− 0.34, 0.50)	0.717	81.6	0.020
Mixed-tocopherols	4	− 0.05 (− 0.40, 0.29)	0.760	10.2	0.342
Dosage of vitamin E (mg/day)					
< 500	12	− 0.14 (− 0.34, 0.06)	0.177	82.0	< 0.001
≥ 500	9	− 0.15 (− 0.37, 0.07)	0.190	52.5	0.032
Health condition					
Healthy	3	0.37 (− 0.05, 0.79)	0.086	0.0	0.387
Unhealthy	18	− 0.22 (− 0.38, − 0.06)	0.008	75.5	< 0.001
Insulin resistance-related disorders	11	− 0.22 (− 0.40, − 0.05)	0.010	67.0	0.001
CVDs	1	0.20 (− 0.30, 0.70)	0.435	–	–
Hemodialysis	5	− 0.04 (− 1.45, 1.38)	0.960	71.8	0.007
Other disease	1	− 3.96 (− 5.51, − 2.41)	< 0.001	–	–
Baseline concentrations of IL-6 (pg/mL)					
Normal (< 4.4 pg/mL)	15	− 0.13 (− 0.28, 0.03)	0.109	66.7	< 0.001
Elevated (≥ 4.4 pg/mL)	6	− 0.50 (− 1.18, 0.18)	0.150	85.6	< 0.001
Adjustment for baseline values					
Adjusted	4	− 0.27 (− 0.56, 0.02)	0.073	53.7	0.090
Non-adjusted	17	− 0.10 (− 0.27, 0.08)	0.264	77.3	< 0.001
**Vitamin E supplementation on serum TNF-α concentrations**					
Overall	19	− 0.03 (− 0.09, 0.02)	0.25	78.9	< 0.001
Intervention duration (week)					
< 8	8	− 0.07 (− 0.13, − 0.01)	0.026	32.0	0.173
≥ 8	11	0.12 (− 0.00, 0.25)	0.052	85.2	< 0.001
Type of vitamin E					
α-Tocopherol	14	0.02 (− 0.06, 0.09)	0.655	82.7	< 0.001
γ-Tocopherol	2	− 0.15 (− 0.29, − 0.01)	0.040	0.0	0.358
Mixed-tocopherols	3	− 0.06 (− 0.15, 0.04)	0.238	57.8	0.093
Dosage of vitamin E (mg/day)					
< 500	11	0.12 (− 0.00, 0.25)	0.052	72.4	< 0.001
≥ 500	8	− 0.07 (− 0.13, − 0.01)	0.027	83.2	< 0.001
Health condition					
Healthy	3	− 0.22 (− 0.46, 0.02)	0.072	39.9	0.189
Unhealthy	16	− 0.02 (− 0.08, 0.03)	0.449	81.1	< 0.001
Insulin resistance-related disorders	13	− 0.02 (− 0.08, 0.03)	0.439	84.8	< 0.001
CVD	1	0.30 (− 0.99, 1.59)	0.650	–	–
Rheumatoid arthritis	2	− 0.04 (− 1.29, 1.21)	0.944	0.0	0.994
Baseline concentrations of TNF-α (pg/mL)					
Normal (< 2.3 pg/mL)	8	− 0.04 (− 0.10, 0.01)	0.125	66.1	0.004
Elevated (≥ 2.3 pg/mL)	11	0.46 (0.10, 0.83)	0.013	82.5	< 0.001
Adjustment for baseline values					
Adjusted	5	− 0.01 (− 0.09, 0.07)	0.820	92.0	< 0.001
Non-adjusted	14	− 0.05 (− 0.12, 0.02)	0.177	62.5	0.001

*WMD* weighted mean difference, *CI* confidence interval, *CRP* C-reactive protein, *IL-6* interleukin-6, *TNF-α* tumor necrosis factor-α.

^a^Obtained from the fixed-effects model.

^b^Refers to the mean (95% CI).

^c^Inconsistency, percentage of variation across studies due to heterogeneity.

^d^Obtained from the Q-test.

**Figure 3:**
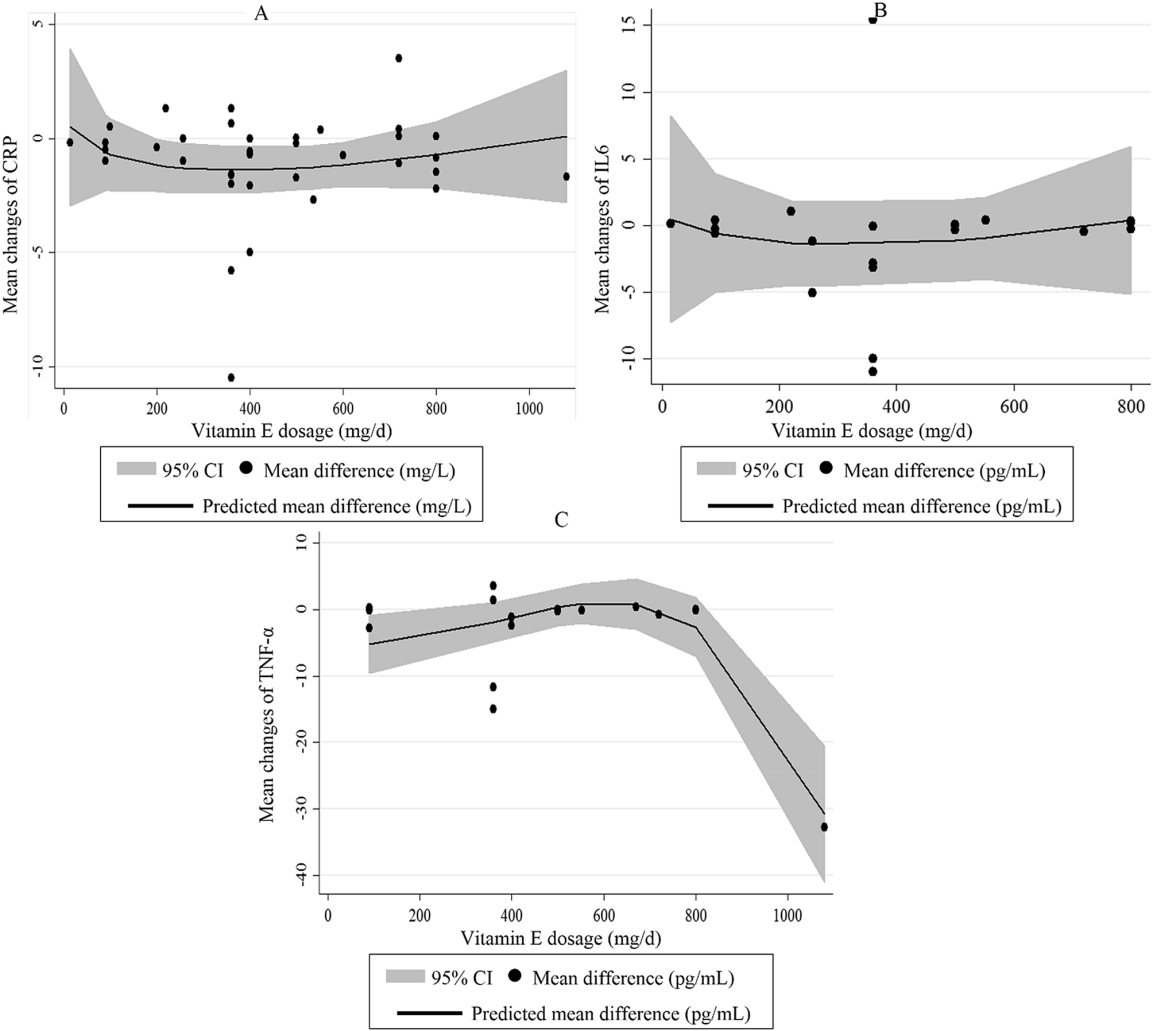
Non-linear dose–response effects of vitamin E dosage (mg/day) on serum concentrations of (**A**) CRP, (**B**) IL-6 and (**C**) TNF-α. The 95% CI is demonstrated in the shaded regions. *CRP* C-reactive protein, *IL-6* interleukin-6, *TNF-α* tumor necrosis factor-α.

The 26 eligible RCTs on serum CRP concentrations were included in the non-linear dose–response meta-analysis. Although not significant, there was a nearly U-shaped curve of the effect of vitamin E dosage on circulating CRP in which the reducing effect of vitamin E gradually increased, and then, the effect gradually decreased and reached to zero value at the dosages of ≥ 1000 mg/day (P_non-linearity_ = 0.39). It seems that the highest reducing effect occurs at the dosages of 300–600 mg/day vitamin E (Fig. 3A). After excluding studies on γ-tocopherol and retaining only those studies that administered α-tocopherol, no change was seen on the non-linear association between vitamin E dosage and changes in CRP levels (P_non-linearity_ = 0.32) (Supplemental Figure 1A).

#### The effect of vitamin E on serum IL-6 concentrations

**Figure 4:**
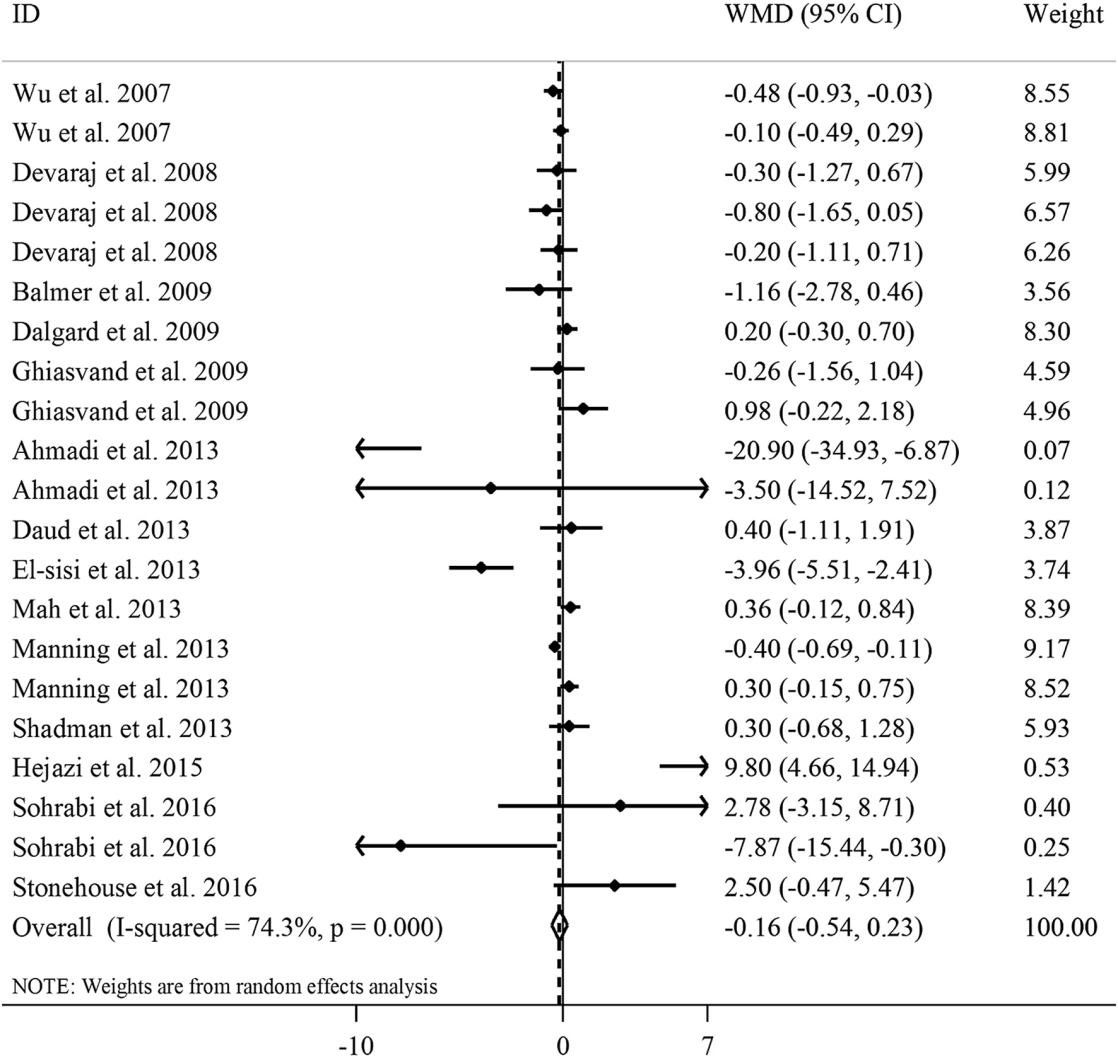
Forest plot for the effect of vitamin E supplementation on serum IL-6 concentrations, expressed as mean differences between intervention and control groups. Horizontal lines represent 95% CIs. Diamonds represent pooled estimates from the random-effects analysis. *IL-6* interleukin-6, *WMD* weighted mean difference, *CI* confidence interval.

In total, 21 effect sizes from 14 RCTs^17,20–23,26–31,33,37,38^, including 902 people, were included in the meta-analysis. Combining the effect sizes, we found no significant effect of vitamin E supplementation on serum IL-6 concentrations (WMD − 0.16, 95% CI − 0.54, 0.23 pg/mL, *P* = 0.42) (Fig. 4).

Heterogeneity between studies was significant in this regard (*I*^2^ = 74.3, P < 0.001). In the subgroup analyses, the type of vitamin E prescribed and participants’ health condition could explain the between-study heterogeneity. Also, vitamin E supplementation resulted in a significant reduction in serum concentrations of IL-6 in RCTs that used α-tocopherol for their intervention and those trials that were performed on unhealthy participants including those with disorders related to insulin resistance. Based on findings from the sensitivity analysis, no single study influenced the overall effect of vitamin E supplementation on serum IL-6 concentrations (range of summary estimates − 0.61, 0.30). Moreover, Begg’s test rejected our hypothesis about the presence of substantial publication bias (P = 0.62). In the non-linear dose–response analysis, we failed to find a significant effect of vitamin E dosage on serum IL-6 concentrations (P_non-linearity_ = 0.57) (Fig. 3B). Such finding was also observed after excluding studies on γ-tocopherol and retaining only those that did supplementation with α-tocopherol (P_non-linearity_ = 0.60) (Supplemental Figure 1B).

### The effect of vitamin E on serum TNF-α concentrations

**Figure 5:**
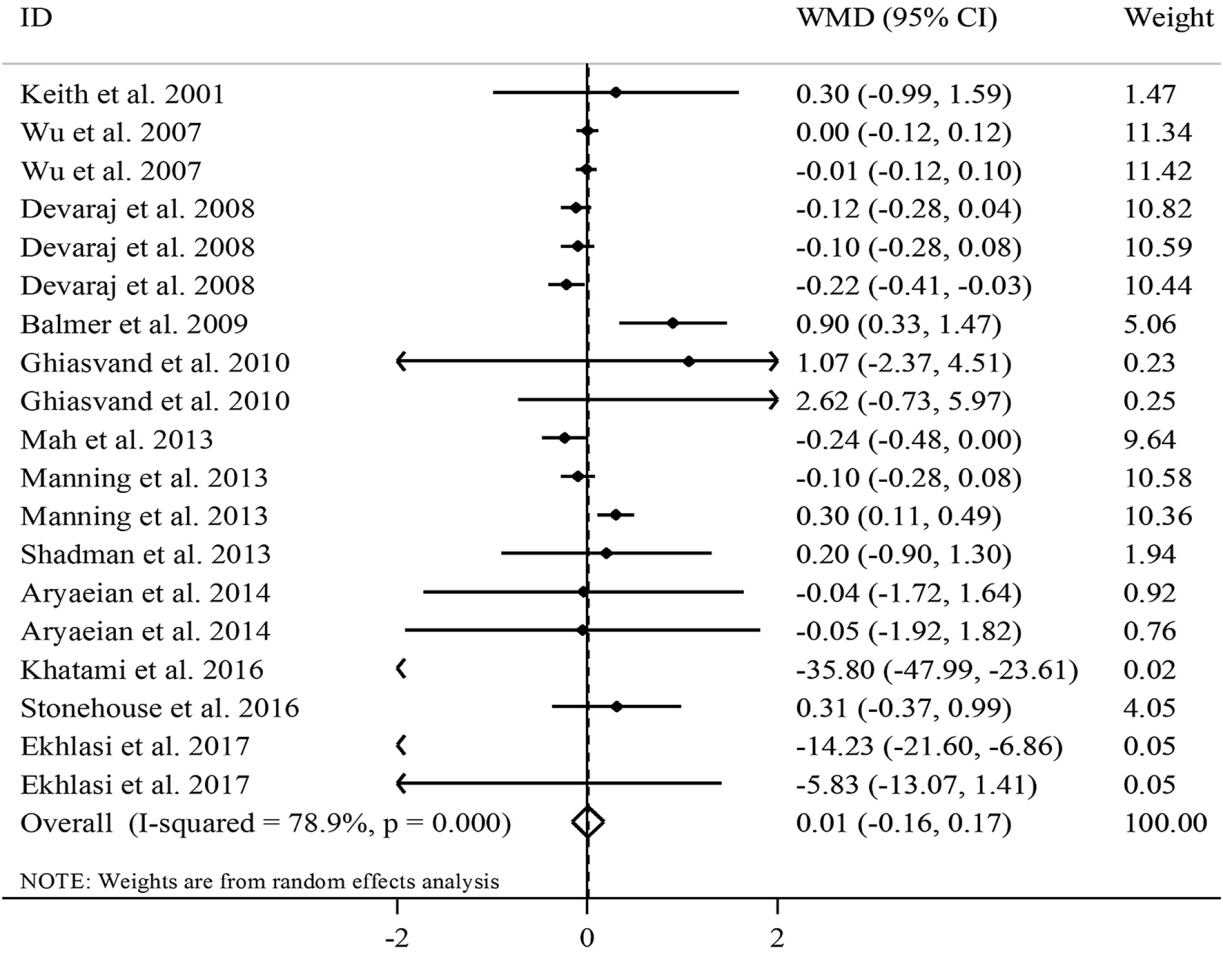
Forest plot for the effect of vitamin E supplementation on serum TNF-α concentrations, expressed as mean differences between intervention and control groups. Horizontal lines represent 95% CIs. Diamonds represent pooled estimates from the random-effects analysis. *TNF-α* tumor necrosis factor-α, *WMD* weighted mean difference, *CI* confidence interval.

Combining 19 effect sizes from 12 RCTs^12,17,20,21,24,29–32,36,38,39^, including a total sample size of 792 participants, we found no significant effect of vitamin E supplementation on serum TNF-α concentrations (WMD − 0.01, 95% CI − 0.16, 0.17 pg/mL, *P* = 0.93) (Fig. 5).

Significant between-study heterogeneity was seen (*I*^2^ = 78.9, P < 0.001). Subgroup analyses according to the duration of intervention, type of vitamin E, and participants’ health condition explained the between-study heterogeneity. From these analyses, we observed that vitamin E supplementation significantly reduced serum TNF-α concentrations at the dosage of ≥ 500 mg/day, in RCTs with a duration of < 8 weeks, and those studies that administered γ-tocopherol rather than other types of tocopherol. Surprisingly, vitamin E supplementation resulted in a significant increase in serum TNF-α concentrations in individuals with elevated levels of TNF-α. Based on the sensitivity analysis, we found that the overall effect size of vitamin E supplementation on serum TNF-α concentrations did not depend on a particular study (range of summary estimates − 0.20, 0.21). In addition, no evidence of substantial publication bias was found based on the Begg test (P = 0.34). Based on the dose–response analysis, we observed a significant non-linear effect of vitamin E dosage on serum concentrations of TNF-α; such that serum TNF-α concentrations were reduced significantly at the dosage of ≥ 700 mg/day vitamin E (P_non-linearity_ = 0.001) (Fig. 3C). When we did dose–response analysis on studies that conducted supplementation with α-tocopherol, the significant effect of vitamin E remained significant (P_non-linearity_ = 0.004) (Supplemental Figure 1C).

## Discussion

In the current meta-analysis, we found that vitamin E supplementation can exert a significant reducing effect, around 0.52 mg/L, on serum levels of CRP in adults. Although this meta-analysis failed to find a significant effect of vitamin E on serum levels of IL-6 overall, there was a significant reducing effect in studies that used α-tocopherol and those that conducted on individuals with disorders associated with insulin resistance. The overall effect size for the effect of vitamin E on serum levels of TNF-α was not significant; however, we observed a significant beneficial effect in studies that used vitamin E at the dosage of ≥ 500 mg/day, those with < 8 weeks’ duration of intervention, and studies that administered vitamin E in the form of γ-tocopherol.

Vitamin E is the most widely studied antioxidant in humans. In vitro studies have revealed a protective role for α-tocopherol in the development of atherosclerotic plaque as on cultured endothelial cells^62^. Indeed, vitamin E inhibits the expression of adhesion molecules stimulated by oxidized low-density lipoprotein (LDL)^63^. Likewise, in vivo animal studies have shown that α-tocopherol can significantly reduce circulating CRP and enhance the scavenging activity of reactive oxygen species (ROSs)^64^. In contrast, results from clinical trials on major cardiovascular properties of vitamin E are not conclusive^65^. In the current meta-analysis, we observed an overall significant reducing effect of vitamin E supplementation in the form of α-tocopherol or mixed isoforms on serum levels of CRP. Similarly, in an earlier meta-analysis of clinical trials, a significant reduction in circulating CRP was observed in patients with hemodialysis following supplementation with vitamin E coated dialyzer^45^. Also, a meta-analysis in 2015 revealed a significant beneficial effect of vitamin E supplements, in the isoforms of α and γ-tocopherols, on serum levels of CRP^44^. That meta-analysis^44^; however, had some limitations which might have distorted the findings. For example, a considerable number of publications^19,20,25,27,30,31,42^ were missed in that study despite meeting the inclusion criteria. Also, the authors excluded studies that used the isoforms of vitamin E other than α- and γ-tocopherols. Despite the conclusions on inflammation, they had only focused on serum levels of CRP.

The reduction of CRP concentrations for an average of 0.52 mg/L following vitamin E in our meta-analysis is an important finding in the clinical setting when compared to lifestyle intervention and bariatric surgery that reduce CRP levels by 0.13 and 0.16 mg/L, respectively^66^. Almost all participants in the studies included in our analysis had a low-grade inflammation (CRP < 10 mg/L); and therefore, 0.5 mg/L reduction in serum CRP is clinically significant in these ranges. Earlier reports found a direct association between CRP concentrations and the risk of CVDs in different populations around the world. In a population-based study, two-fold higher mortality from CVDs was reported in serum CRP of > 3 mg/L, while the optimal level confirmed by the AHA and the CDC is < 1 mg/L^67^. In the Women's Health Study, by increasing quintiles of CRP (≤ 0.49, > 0.49 to 1.08, > 1.08 to 2.09, > 2.09 to 4.19, and > 4.19 mg/L), the corresponding relative risks of a first cardiovascular event were 1.0, 1.4, 1.6, 2.0, and 2.3 (P for trend < 0.001), in the adjusted model^68^. Most interventions with reducing CVD risk have been linked to lower CRP levels^69^; however, definitive evidence that lowering CRP levels will necessarily result in reduced cardiovascular events is lacking. Besides, despite well-documented beneficial effects of vitamin E on inflammation, as well as the inverse relationship between vitamin E consumption and the risk of chronic diseases, many large intervention studies have failed to support consistent benefits of vitamin E for the prevention of chronic diseases such as cancer and CVDs^70,71^.

The measurement of CRP is a powerful predictor for cardiovascular mortality^72^. However, given the high fluctuations upon inflammation or even health status, CRP measurement alone is not enough to represent the immunomodulatory changes^73,74^. Therefore, there is a need for evaluating other biomarkers of subclinical inflammation. In our analysis, unlike serum CRP concentrations, the overall effect of vitamin E on circulating IL-6 and TNF-α was not significant. Similarly, in a population-based cohort study, the intake of α-tocopherol was negatively associated with serum levels of CRP but not IL-6 after adjustment for confounding variables (65). However, this finding is not consistent with that of the previous meta-analysis on hemodialysis patients that suggested a significant reduction in circulating IL-6 following supplementation with vitamin E coated dialyzer^45^.

When we did subgroup analyses, we found a significant reducing effect of vitamin E on serum levels of IL-6 and CRP in studies performed on subjects with insulin resistance-related disorders including type 2 diabetes, metabolic syndrome, and non-alcoholic fatty liver disease. These metabolic conditions are associated with several pathophysiological problems resulting in elevated baseline levels of inflammatory biomarkers^75^. Since elevated levels of inflammatory biomarkers are more sensitive to the supplementation of antioxidants^76^, it may explain our findings in this subgroup.

We also found a non-significant U-shape dose–response effect of vitamin E (or α-tocopherol) dosage on the reduction of serum CRP in which the effect had a gradually increasing trend from 0 to 400 mg/day and then it had a decreasing trend from 400 to 1000 mg/day; such that at the dosages of ≥ 1000 mg/day, vitamin E had no significant effect on serum CRP concentrations. It seems that the highest reducing effect occurs at the dosages of 300–600 mg/day. This is consistent with the recommended dietary allowance (RDA) and tolerable upper intake level (UL) of vitamin E for adults that was suggested to be 15 mg (22.4 IU) and 1000 mg α-tocopherol (1500 IU), respectively^77^. Antioxidants at high doses, not only act as prooxidants but also disrupt redox balance through interaction with physiological concentrations of ROS required for optimal cellular functioning resulting in cellular dysfunction^78^. In the subgroup meta-analysis, we observed a significant reduction in serum levels of CRP and TNF-α in studies that used vitamin E at the dosage of ≥ 500 mg/day. Likewise, our dose–response meta-analysis revealed a significant reduction in serum TNF-α at the dosage of ≥ 700 mg/day vitamin E. This finding; however, contradicts the earlier meta-analysis in which the dosages lower than 400 IU/day significantly reduced circulating CRP^44^. Findings from a dose–response meta-analysis revealed that a minimum dosage of 400 IU/day (266 mg/day) α-tocopherol is required for the reduction of LDL^79^. Therefore, smaller dosages of vitamin E may not sufficient to exert anti-inflammatory effect; however, higher dosages of vitamin E should be used cautiously due to the probability of increased risk of all-cause mortality^80^.

We also found a significant decline in CRP concentrations in studies with a long duration of the intervention (≥ 8 weeks). This would seem to oppose our findings of TNF-α, in which the beneficial effect was seen for studies with a short duration of the intervention (< 8 weeks). The contradictory findings of CRP and TNF-α might be explained by the fact that most studies that assessed TNF-α levels did not control the analyses for baseline values of this biomarker^12,20,21,24,29–32,38^, while almost all the included RCTs on circulating CRP performed adjustment for baseline concentrations of this biomarker. Furthermore, the lack of a significant effect on TNF-α in studies with longer duration of follow-up might be due to selecting patients with well-controlled disease conditions resulting in a normal level of TNF-α.

Considering different isoforms of vitamin E, we found that only α-tocopherol significantly reduced serum levels of CRP and IL-6, whereas the effect of γ-tocopherol was not significant.

Both isoforms are the most prevalent forms of vitamin E in diets, supplements, and tissues; however, the levels of α-tocopherol are approximately tenfold higher than γ-tocopherol in tissues due to the preferential transfer of α-tocopherol to lipid particles^81^. Therefore, α-tocopherol is more likely to potentiate ROS scavenging than γ-tocopherol. Besides, in our analysis, combined administration of both α- and γ-tocopherol reduced serum levels of CRP. Unlike earlier reviews with reports of opposing regulatory function of different isoforms of vitamin E for inflammation^82^, recent preclinical and clinical studies have shown that different forms of vitamin E have unique anti-inflammatory properties^83^. Moreover, some clinical trials revealed that supplementation with the α-tocopherol isoform of vitamin E can reduce plasma γ-tocopherol^84^. This may happen as a result of the function of hepatic α-tocopherol transfer protein (α-TTP)^85^. α-TTP, together with ATP-binding cassette transporter A1 (ABCA1), potentially incorporates α-tocopherol into the plasma as well as increasing γ-tocopherol metabolism^86,87^. However, recent studies have rejected the decreased levels of γ-tocopherol induced by α-tocopherol supplementation when they were supplemented in combination^20^. Thus, there may be benefits in avoidance of potential adverse effects following supplementation with both isoforms.

Considering the above-mentioned points, it seems that the different responses of CRP, IL6, and TNF-α to vitamin E depend on the isoforms of this vitamin, dosage, duration of consumption, and health condition of consumers. For instance, CRP and IL6 levels were reduced by supplementation with α-tocopherol, particularly in subjects with an unhealthy condition such as those with insulin resistance-related disorders, while TNF-α levels were reduced by γ-tocopherol supplementation, or both CRP and TNF-α responded to high dosages of vitamin E (≥ 500 mg/day). Further studies are needed to examine the effect of vitamin E supplementation on other inflammatory biomarkers in adults.

Mechanistic evidence for the anti-inflammatory properties of α-tocopherol is not completely discovered. This tocopherol reduces the production of ROS from monocytes through activating the activation protein 1 (AP-1), which can dephosphorylate protein kinase C (PKC) and inhibit the proliferation of smooth muscle cells^88^. ROSs are important free radical species that cause endothelial dysfunction by altering cell membrane integrity and subsequent cell death^89^. Also, ROSs play an important role in increasing the concentrations of inflammatory cytokines^90^. Moreover, in different cell types, vitamin E may induce an anti-inflammatory effect through inhibiting the COX-2 and 5-LUX mediated eicosanoids and suppressing the NF-κB and JAK-STAT6 or JAK-STAT3 signaling pathways^83^.

In the current meta-analysis, we gathered all available evidence about the effect of all types of supplemental vitamin E on serum concentrations of inflammatory biomarkers. However, some potential limitations should be addressed when interpreting our findings. There was considerable heterogeneity between the included studies. In the subgroup analysis, type and dosage of tocopherol, participants’ health condition, and baseline serum concentrations of inflammatory cytokines could explain the variation between studies. Moreover, we could not determine a safety margin of supplemental vitamin E due to the lack of serious adverse events in the selected studies. High-dose vitamin E has been debated for the safety such that the dosages of > 400 IU/day have been reported to be associated with increased all-cause mortality by increasing the risk of prolonged bleeding time^80^. However, other meta-analyses did not report this increase in total mortality^91,92^. Moreover, some included studies were conducted on patients with different metabolic diseases; and therefore, caution should be taken when extrapolating our findings to the general population. Last but not least, since most studies did not report serum levels of α-tocopherol, it is not clear whether the effect is dependent on the status of serum vitamin E.

Altogether, the results of this meta-analysis are in favor of a CRP-reducing effect of supplementation with α-tocopherol, especially at the dosage of ≥ 500 mg/day, alone or in combination with γ-tocopherol. Higher dosages of vitamin E (> 1000 mg/day) are not effective in the reduction of subclinical inflammation; and therefore, are not recommended. Furthermore, a significant reduction in serum levels of IL-6 was also seen in studies that used α-tocopherol. Despite the confirmed health effects of vitamin E, literature supporting actual clinical benefits on hard endpoint including morbidity or mortality caused by just reduction in serum concentration of CRP is disappointing. Thus, the accumulating evidence on the anti-inflammatory properties of tocotrienol warrants future research on the clinical setting and larger population. Moreover, since there may be benefits in avoidance of potential adverse effects following supplementation with both isoforms, future studies are needed to further examine this issue.

## Supplementary information

Supplementary Information.

## Abbreviations

CRP: C-reactive protein
IL-6: Interleukin 6
TNF-α: Tumor necrosis factor-α
DM: Diabetes mellitus
CVD: Cardiovascular disease
NASH: Nonalcoholic steatohepatitis
NAFLD: Non-alcoholic fatty liver disease
HD: Hemodialysis
MetS: Metabolic syndrome
RCT: Randomized clinical trial
