# Supplementary material for: The effect of vitamin E supplementation on selected inflammatory biomarkers in adults: a systematic review and meta-analysis of randomized clinical trials
Source: Sci Rep. 2020 Oct 14;10:17234. doi: 10.1038/s41598-020-73741-6 (PMC7560744; doi:10.1038/s41598-020-73741-6)
Supplement: Supplementary file 1 — Supplementary Information. [file 41598_2020_73741_MOESM1_ESM.docx]

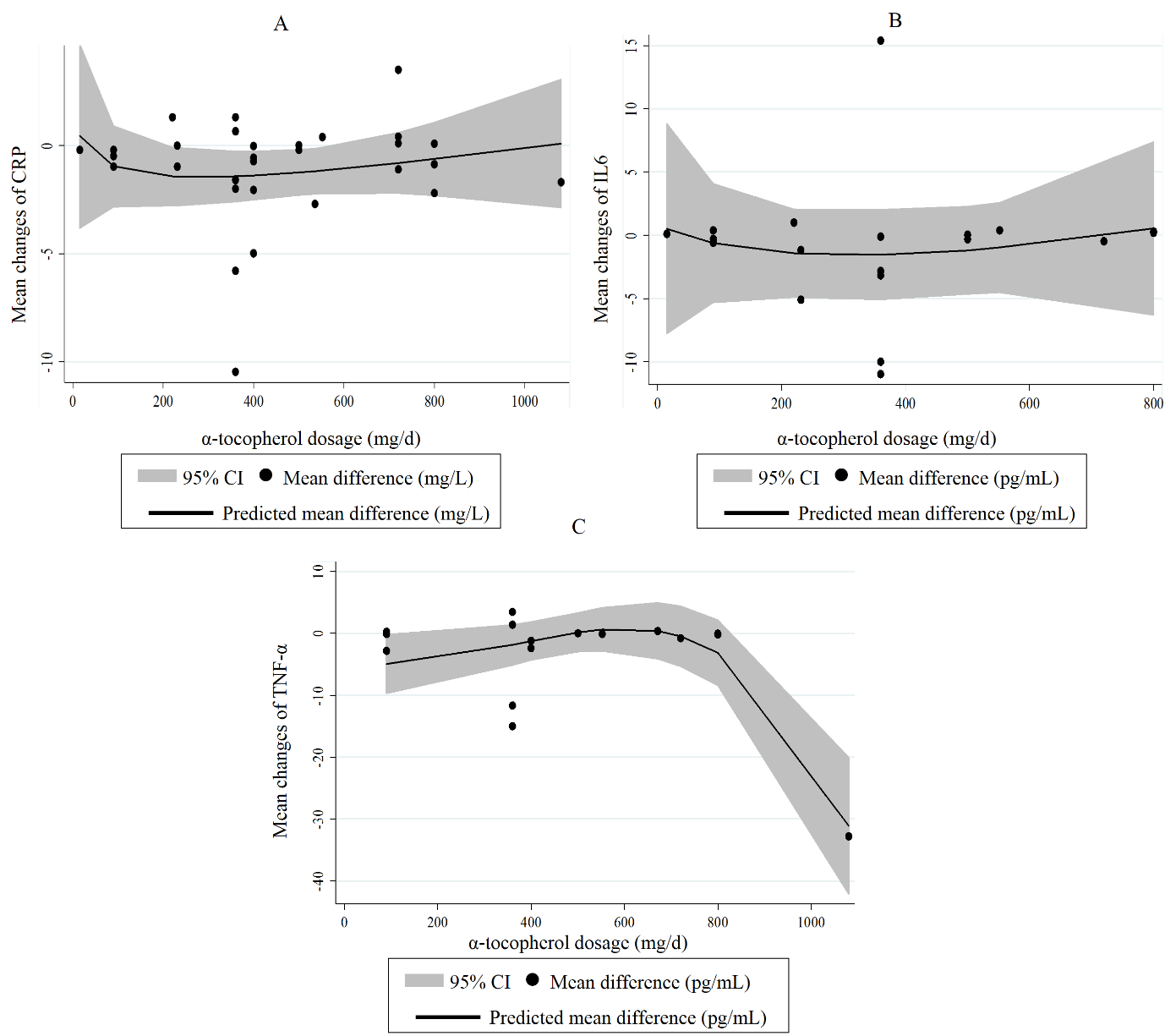


**Supplemental Figure 1:** Non-linear dose-response effects of α-tocopherol dosage (mg/d) on serum concentrations of (A) CRP, (B) IL-6 and (C) TNF-α. The 95% CI is demonstrated in the shaded regions. CRP: C-reactive protein, IL-6: interleukin-6, TNF-α: tumor necrosis factor-α.

**Supplemental Table 1**: Results of risk of bias assessment for randomized clinical trials included in the current meta-analysis on the effects of vitamin E supplementation on inflammatory biomarkers^1^

| Study | Random Sequence Generation | Allocation concealment | Reporting bias | Other sources of bias | Performance bias | Detection bias | Attrition bias |
| --- | --- | --- | --- | --- | --- | --- | --- |
| Upritchard et al. 2000 | L | U | H | H | U | U | L |
| Keith et al. 2001 | L | L | H | H | L | L | L |
| Murphy et al. 2004 | L | U | H | H | L | U | L |
| Lopez et al. 2004 | L | L | H | L | L | U | L |
| Hodkova et al. 2006 | L | L | H | H | U | H | L |
| Singh et al. 2007 | L | H | H | H | L | U | L |
| Wu et al. 2007 | L | U | L | H | L | U | L |
| Aryaeian et al. 2008 | L | H | H | L | L | H | L |
| Castilla et al. 2008 | L | H | H | L | H | H | L |
| Devaraj et al. 2008 | L | H | L | H | L | H | L |
| Balmer et al. 2009 | L | U | H | H | L | L | L |
| Dalgard et al. 2009 | L | U | H | L | L | H | L |
| Ghiasvand et al. 2009 | L | H | H | H | L | H | L |
| Ghiasvand et al. 2010 | L | H | H | H | L | H | L |
| Rafraf et al. 2012 | L | U | H | L | L | H | L |
| Ahmadi et al. 2013 | L | H | H | L | L | H | L |
| Daud et al. 2013 | L | L | H | L | L | L | L |
| El-sisi et al. 2013 | L | H | H | H | L | H | L |
| Mah et al. 2013 | L | U | L | L | L | U | L |
| Manning et al. 2013 | L | L | L | L | L | L | L |
| Shadman et al. 2013 | L | H | L | L | L | H | L |
| Aryaeian et al. 2014 | L | U | H | L | L | U | L |
| Gopalan et al. 2014 | L | L | H | H | L | L | L |
| Hejazi et al. 2015 | L | H | H | H | L | H | L |
| Modi et al. 2015 | L | H | H | H | L | H | L |
| Ramezani et al. 2015 | L | U | H | L | L | H | L |
| Khatami et al. 2016 | L | L | H | L | L | H | L |
| Sohrabi et al. 2016 | L | L | H | L | H | H | L |
| Stonehouse et al. 2016 | L | U | L | L | L | U | L |
| Ekhlasi et al. 2017 | L | L | H | L | L | L | L |
| Pervez et al. 2018 | L | U | H | H | L | U | L |
| Devaraj et al. 2007 | L | H | L | H | L | U | L |
| Rachelle et al. 2011 | L | U | H | H | L | U | L |

^1^Each study was assessed for risk of bias using the Cochrane Risk of Bias Assessment tool (Ref. 45). Domains of assessment were included random sequence generation, allocation concealment, reporting bias, performance bias, detection bias, attrition bias and other sources of bias. Each domain was scored as “high risk” if it contained methodological flaws that may have affected the results, “low risk” if the flaw was deemed inconsequential, and “unclear risk” if information was insufficient to determine. If a study got “low risk” for all domains, it considered as a high quality study with totally low risk of bias.
